# Comparative Real-World Analysis of Baseline Demographic Characteristics and Comorbidities in Atopic Dermatitis Patients Initiating Biologics Versus JAK Inhibitors

**DOI:** 10.3390/jcm14041291

**Published:** 2025-02-15

**Authors:** Alvaro Prados-Carmona, Francisco J. Navarro-Triviño, Husein Husein-ElAhmed, Ricardo Ruiz-Villaverde

**Affiliations:** 1 Department of Dermatology, Hospital Universitario San Cecilio, 18016 Granada, Spain; fntmed@gmail.com; 2 Instituto Biosanitario de Granada, Ibs, 18001 Granada, Spain; huseinelahmed@hotmail.com; 3 Escuela Internacional de Posgrado, Universidad de Granada, 18001 Granada, Spain; 4 Department of Contact Eczema and Immunoallergic Diseases, Dermatology, Hospital Universitario San Cecilio, 18007 Granada, Spain; 5 Department of Dermatology, Hospital Universitario de Baza, 18800 Granada, Spain

**Keywords:** atopic dermatitis, systemic advanced therapies, biologic drugs, JAK inhibitors, treatment, real-world data, comparative analysis

## Abstract

**Background**: Systemic advanced therapies, including biologic drugs and Janus kinase (JAK) inhibitors, have revolutionized atopic dermatitis management. The increasing number of available options for such complex diseases demands careful treatment selection for each patient, considering numerous variables. Comparative analyses of these treatment modalities in the real world are still limited. Only a faithful basal characterization would enable posterior meaningful and accurate comparisons of the efficacy and safety profiles of these groups of drugs. This communication focuses on describing and comparing the baseline demographics and comorbidities of patients with atopic dermatitis currently treated with biologic therapies versus JAK inhibitors in our setting. **Methods**: We conducted an observational, descriptive, and ambispective study across three hospitals covering a population of over 500,000 inhabitants from January 2019 to December 2024. Baseline demographic data, anthropometric measures, lifestyle factors, cardiovascular risk factors, and comorbidities were analyzed using descriptive and inferential statistics. Additionally, basal severity and effectivity over time have also been compared. **Results**: A total of 150 patients were analyzed. A total of 102 had received biological therapies (dupilumab or tralokinumab), whereas 48 patients had received JAK inhibitors (upadacitinib, baricitinib, or abrocitinib). Ages ranged from 11 to 76 years. The overall cohort had a mean age of 35.87 ± 14.37 years and a male predominance (male-to-female ratio 1.63:1). Hypertension was more prevalent in the JAK inhibitors group (*p* = 0.0175), yet other cardiovascular risk factors, body measurements, atopic and non-atopic comorbidities, and disease severity were comparable across both groups. **Conclusions**: This study helped to characterize the baseline characteristics of patients treated with advanced systemic therapies in a real-world clinical setting. It pointed to just slight differences between the profiles of patients treated with biologics versus JAK inhibitors. This homogeneity in baseline characteristics sets the ground for further future comparisons of treatment outcomes in this cohort as potential confounding factors related to group imbalances are minimized.

## 1. Introduction

Atopic dermatitis (AD) is a chronic, relapsing inflammatory skin disease characterized by intense pruritus, eczematous lesions and a significant impact on patients’ quality of life [1]. It is one of the most common dermatological conditions worldwide, affecting up to 20% of children and 10% of adults [1,2], with marked geographical variation in prevalence [3,4,5,6,7]. The pathophysiology of AD is complex, involving environmental factors and genetic predispositions to skin barrier dysfunction and immune dysregulation, underscoring its potential heterogeneity [8,9,10,11,12]. Beyond its cutaneous manifestations, AD is associated with a wide spectrum of comorbidities, reflecting its systemic nature. Patients frequently exhibit allergic comorbidities such as asthma, allergic rhinitis, and food allergies, alongside non-atopic conditions, including cardiovascular disease, obesity, and psychiatric disorders [10,13,14,15,16,17,18,19,20,21,22,23,24,25]. The chronic inflammatory milieu inherent in AD is thought to contribute to this broad range of comorbidities. This introduces additional complexity to managing AD, particularly in patients requiring systemic therapy, highlighting the need for a tailored approach to treatment selection that considers the patient’s overall health profile.

Over the last decade, the therapeutic landscape for AD has undergone a transformative shift with the development of systemic advanced therapies, particularly biologic agents and Janus kinase (JAK) inhibitors, offering targeted treatment modalities that have redefined disease management [8,26,27,28,29,30,31,32]. However, their introduction also presents new challenges in clinical decision-making due to their higher costs and the complexity of treatment selection. Clinicians must consider numerous factors, including patient demographics, comorbidities, and overall health, when choosing between these therapies. As systemic advanced therapies are significantly more expensive than conventional immunosuppressants, ensuring their optimal use is critical to balancing healthcare efficiency with clinical outcomes.

Randomized controlled trials (RCTs) remain the gold standard for establishing the efficacy and safety of new therapies. Although they provide robust evidence under controlled conditions, their external validity—reflecting the applicability of results to broader, more diverse patient populations—often remains to be fully elucidated after the drug’s approval. Real-world data (RWD), derived from routine clinical practice, helps bridge the remaining gap by offering insights into how treatments perform in heterogeneous, unselected patient groups, including those with comorbidities and complex medical histories typically excluded from RCTs [33]. It does not substitute but complements RCT evidence by capturing patient outcomes in routine clinical practice, reflecting the diversity and challenges encountered in real-life settings. Therefore, it has emerged as an essential tool for clinicians to navigate the decision-making process for treatment selection in dermatology. Indeed, RWD has already been pivotal in guiding therapeutic strategies for other chronic inflammatory conditions with a wide range of systemic treatment options, such as psoriasis, where its role in understanding treatment patterns and optimizing resource allocation is well established [33,34,35,36]. In contrast, RWD is only beginning to emerge in AD, which has a shorter trajectory in terms of advanced therapeutic options [8,37,38]. Nevertheless, the field is rapidly evolving, and the increasing availability of approved therapies, together with a constantly expanding pipeline, underscores the importance of robust real-world comparisons to ensure optimal use of resources while delivering personalized care [39,40,41,42]. This is particularly significant in AD as it has a high placebo response rate, as seen in many of the already available clinical trials [2,43,44,45,46]. To date, comprehensive comparative analyses of the use of biologics and JAK inhibitors in real-world settings are still lacking [47,48,49]. Populations enrolled in clinical trials are carefully selected and tend to be similar to one another and might allow indirect comparisons of drugs within the controlled conditions of these studies. However, such homogeneity does not reflect the complexities and variability of patients seen in routine clinical practice. Comparisons between each drug RWD and prior RCT results, and between different drugs in real-world settings, are largely dependent on an adequate characterization of the profile of patients receiving the drug after approval [47,50,51]. Therefore, it is critical to understand how these treatments are currently being used in real-world practice to, firstly, enable faithful comparisons between them and, secondly, elucidate whether the ongoing strategies align with the goal of maximizing both clinical outcomes and healthcare efficiency. So far, the different, if existing, profiles of patients currently receiving the different treatment modalities, biologics, or JAK inhibitors in clinical practice have not been thoroughly delineated yet.

In this work, we intend to shed light on the baseline profiles and the reasons why clinicians select specific therapies in our setting. We believe this is a critical step toward enabling meaningful and accurate comparisons of the efficacy and safety profiles of these advanced therapies in real-world settings. Many factors, such as patient demographics, overall health status and comorbidities, and disease-related variables, should be explored. This study specifically aims to evaluate and compare the baseline demographic and clinical characteristics outside of AD in patients initiating biologics versus those starting JAK inhibitors in a healthcare area serving more than 500,000 inhabitants over a six-year period. These advanced systemic treatments have been prescribed within the Spanish National Health System under unique guidelines (patients with moderate-to-severe AD that have not achieved adequate control with at least one prior conventional systemic treatment). In the region studied, these therapies are centralized in the involved institutions, so only a few specialized dermatologists with a unified treatment protocol were in charge of their prescriptions, ensuring the avoidance of potential biases or differences in criteria. Additionally, we will also analyze differences in basal disease severity and effectivity over time between groups. This will be done using widely adopted scales such as Eczema Area and Severity Index (EASI), SCORing Atopic Dermatitis (SCORAD), Dermatology Life Quality Index (DLQI), and Patient-Oriented Eczema Measure (POEM). Follow-up studies will complement this information with other important baseline determinants of treatment selection, including a proper in-depth analysis of additional disease-related aspects (family and personal history of AD, age of onset, emollient use, prior treatments received, frequency of emergency room visits, hospitalizations related to AD, disease clinical pattern) and laboratory findings. Our main goal is to assess the homogeneity and comparability between patients treated with biologics or JAK inhibitors in our area, with a special focus on basal demographics and health status outside AD. By identifying heterogeneously distributed factors, we could provide insights and hypothesize about those that might have influenced therapeutic allocation in real-world practice. This would settle an adequate foundation for subsequent comparisons among drugs and ultimately contribute to the growing body of evidence that supports personalized medicine in AD management.

**Bulleted summary:**

- RWD is indispensable for providing a more comprehensive understanding of treatment outcomes. Therefore, it is also emerging as an essential tool for treatment selection in the heterogeneous populations encountered in daily clinical practice.
- RWD comparisons of systemic advanced therapies require prior adequate characterization of the profile of patients receiving the drugs, ensuring homogeneity to be meaningful and reliable for decision-making. If there were different profiles of patients receiving the different drugs, outcome comparisons would need to take into account those profile-dependent characteristics to avoid misleading conclusions.
- This study aims to evaluate and compare the baseline demographic and clinical characteristics outside AD of AD patients initiating biologics versus those starting JAK inhibitors across three hospitals covering a population of over 500,000 inhabitants over a six-year period.

## 2. Materials and Methods

We conducted an observational, descriptive, and ambispective study across three hospitals from the same province in southern Spain: Hospital Universitario San Cecilio, Granada (serving a population of approximately 480,000 people); Hospital Santa Ana, Motril (approximately 140,000 people); and Hospital de Baza (approximately 65,000 people). The study aimed to evaluate and compare the baseline demographic and clinical characteristics of patients with AD receiving systemic advanced therapies, including biologic agents and JAK inhibitors, between January 2019 and December 2024.

Study Population and Patient Selection: The study population comprised patients with a confirmed diagnosis of moderate-to-severe AD eligible for treatment at any time of the study period. Patients were identified retrospectively through the hospital’s electronic medical records system and prospectively during their consultation in the dermatology department.

The inclusion criteria were:

(a) Age ≥ 6 years.
(b) Diagnosis of AD based on established criteria, including Hanifin and Rajka, UK Working Party, or subsequent adaptations for use in the clinical setting [52,53,54,55,56].
(c) Initiation of systemic advanced therapy with either a biologic agent (Group A: dupilumab, tralokinumab) or a JAK inhibitor (Group B: upadacitinib, baricitinib, or abrocitinib).
(d) Availability of enough baseline data for demographic and clinical characterization.
(e) Provision of written informed consent for the use of their medical data for research purposes (or consent from relevant legal guardian).

The exclusion criteria were:

(a) Patients who did not consent to participate in the study.
(b) Incomplete or missing medical records for baseline characteristics.
(c) Use of both biological therapies and JAK inhibitors concurrently.
(d) AD patients receiving advanced systemic therapy for off-label indications unrelated to AD.
(e) Coexistence of data suggesting an alternative dermatological or non-dermatological diagnosis that justifies signs (e.g., eczema) or symptoms (e.g., pruritus) overlapping with those of the disease under study.
(f) Pregnancy, breastfeeding, or other known contraindications for the use of the drugs under study.
(g) Evidence of adherence problems to previous treatments.

Variables assessed: Data collected included patient demographics (sex, age, and residential location), body measures (weight, height, body mass index [BMI]), and medical history (habits, cardiovascular risk factors, atopic and non-atopic comorbidities). Additionally, basal severity scores (EASI, SCORAD, DLQI, POEM) and efficacy over time were also introduced to comply with the reviewing request. Further disease-related data were specifically excluded to ensure a targeted analysis of the sociodemographic, anthropometric, and general health-related variables considered here.

Data collection, analysis, and manuscript writing: Data for this study were obtained using a combination of retrospective and prospective methods. Retrospective data were extracted from the hospital’s electronic medical records system. The index date for each patient was defined as the date of prescription of systemic advanced therapy (either biologic or JAK inhibitor). Baseline data were defined as variables recorded within 6 months prior to or on the index date. Prospectively, information was collected using ad-hoc designed forms. These forms were specifically developed for this study and completed during routine dermatological care with the collaboration and consent of the participants. Descriptive and inferential statistics were used to summarize and compare baseline characteristics between Group A and Group B. Measures of central tendency have been reported alongside their corresponding measures of dispersion (e.g., mean [± standard deviation]). Where relevant, minimum and maximum values have also been provided. Categorical variables were presented as frequencies and percentages. Group comparisons were conducted using Student’s *t*-test or Mann–Whitney U test for continuous variables, as appropriate. The chi-square test or Fisher’s exact test was used for categorical variables. Confidence intervals have been estimated at the 95% level, and all statistical tests were two-sided, with significance set at *p* < 0.05. All analyses were conducted using SPSS v.25 (SPSS, Chicago, IL, USA). During the preparation of this work, the authors used ChatGPT-4.0 (OpenAI, San Francisco, CA, USA) with human oversight to improve the readability of parts of the work. After using this tool, the authors reviewed and edited the content as needed and took full responsibility for the content of the publication.

Ethical considerations: The study was conducted in accordance with the Declaration of Helsinki and approved by the Institutional Review Board of Hospital Universitario San Cecilio, Granada, Spain (protocol code FIB-DAT-2024-20 v1.1, date of approval 31 October 2024).

## 3. Results

### 3.1. Participant Demographics and Anthropometric Measurements

The study included 150 patients with moderate-to-severe AD, of whom 102 (68%) initiated treatment with biologic therapies (Group 1), while 48 (32%) were treated with JAK inhibitors (Group 2). The overall cohort had a male predominance, with a male-to-female ratio of 1.63:1. In Group 1, 62 males (60.8%) and 40 females (39.2%) were treated, whereas Group 2 comprised 31 males (64.6%) and 17 females (35.4%), with no significant differences in sex distribution between the groups (*p* = 0.8961).

The mean age of the overall cohort was 35.87 ± 14.37 years, ranging from 11 to 76 years. Group 2 patients were slightly older on average (37.00 ± 12.56 years) compared to Group 1 (35.33 ± 15.07 years), though this difference was not statistically significant (*p* = 0.3627). Among the 150 participants, 8 were under 18 years of age, with 7 in Group 1 and 1 in Group 2 (*p* = 0.0671).

Anthropometric measurements showed statistically significant differences in height (168.44 cm vs. 173.71 cm, *p* = 0.0066) and weight (71.27 kg vs. 77.34 kg, *p* = 0.0387) between the groups. However, no significant differences were observed in BMI, mean 25.24 ± 4.20 kg/m^2^ (25.08 kg/m^2^ vs. 25.59 kg/m^2^, *p* = 0.51), or abdominal perimeter, mean 88.99 ± 13.76 cm (88.58 cm vs. 89.92 cm, *p* = 0.5794) across all patients; see Table 1.

### 3.2. Lifestyle and Cardiovascular Risk Factors

Lifestyle factors, including residential area type, physical activity, tobacco use, and alcohol consumption, were analyzed for both groups. A total of 69 patients lived in big urban areas, 67 in smaller villages closer to rural environments, and only 14 in settlements near the coast. There were no statistically significant differences in residential zone distribution between Group 1 and Group 2 (*p* = 0.1176). A similar proportion of patients reported engaging in regular physical exercise (*p* = 0.5261). The average number of exercise sessions per week was 2.5 ± 2.06 across the sample. Tobacco use, classified as current, former, or never smokers, did not significantly differ between the groups (*p* = 0.4562), nor did alcohol intake (*p* = 0.1761).

Cardiovascular risk factors were evaluated, with hypertension being significantly more prevalent among patients in Group 2 compared to Group 1 (*p* = 0.0175). However, no significant differences were found for other risk factors, including diabetes mellitus (*p* = 0.4744), dyslipidemia (*p* = 0.7723), and metabolic syndrome (*p* = 0.604). Chronic kidney disease, another factor influencing classic immunosuppressive treatment decisions in AD patients, showed no significant variation between the groups (*p* = 0.6864). See Table 2.

### 3.3. Atopic and Other Non-Atopic Comorbidities

The prevalence of atopic comorbidities, such as asthma and allergic rhinoconjunctivitis, was similar across both groups. Asthma was present in 44.66% of the overall cohort, with no significant differences between Group 1 and Group 2 (*p* = 0.2144). Rhinoconjunctivitis was reported in 66.66% of patients, with the same frequency in both groups (*p* = 1.0). Allergic sensitization, assessed through exposure to pneumallergens and trophoallergens, also showed no significant differences between the treatment groups (*p* = 0.9962 and *p* = 0.1904, respectively). The prevalence of allergic contact dermatitis was similar in both groups (*p* = 0.6669).

Other comorbidities not traditionally linked to the so-called “atopic-march” were also evaluated. Immune-mediated diseases (IMIDs), including psoriasis, alopecia areata, and celiac disease, did not significantly differ between the groups (*p* = 0.601, *p* = 0.4881, and *p* = 1, respectively). Gastroesophageal reflux, reported by a minority of patients, showed no significant difference between the two groups (*p* = 0.114). Similarly, there were no differences in patients with prior varicella-zoster virus (VZV) infection or reactivation (*p* = 0.7682), those with a history of neoplasms (*p* = 1.0), or with prior ophthalmologic disease including red eye (*p* = 0.9233). See Table 2.

### 3.4. Severity Scales and Treatment Response

The baseline scores for key severity and quality-of-life scales, including EASI, SCORAD, DLQI, and POEM, were comparable between the biologic and JAK inhibitor treatment groups. This similarity indicates that patients in both cohorts presented with a consistent level of disease severity and impact on quality of life at the start of the study. Comparisons over time and between groups at baseline and after 16 weeks of treatment are shown in Table 3.

### 3.5. Summary of Key Findings

Both groups had a male predominance and similar age distributions, with no statistically significant differences in sex ratio or mean age. However, only one patient out of eight under 18 years of age was treated with JAK inhibitors. Despite differences in height and weight, BMI and abdominal perimeter did not differ significantly between groups. Tobacco use, alcohol intake, area of residence, and physical activity were also similar across groups. Hypertension was significantly more prevalent in Group 2 (*p* = 0.0175), whereas other cardiovascular risk factors, such as diabetes, dyslipidemia, and metabolic syndrome, were comparable between the groups. No significant differences were observed in the prevalence of atopic conditions (e.g., asthma, rhinitis) or non-atopic comorbidities (e.g., psoriasis, alopecia areata…). Baseline scores for key severity and quality-of-life scales were also comparable. However, those severity scales that included subjective patient reports showed differences at week 16 between groups.

## 4. Discussion

This study provides a comparative analysis of key baseline characteristics of patients with moderate-to-severe AD initiating biologic therapies versus JAK inhibitors. Demographics and comorbidities have been evaluated in a cohort of 150 patients treated in a real-world setting. This research has served to evaluate trends in treatment allocation and underscores the homogeneity of patients receiving these advanced therapies in our area of influence, a critical step for warranting RWD comparisons among drugs.

AD typically showcases a higher prevalence in females during adolescence and adulthood [57,58,59]. By contrast, our cohort demonstrated a male predominance without significant differences between groups, but this is consistent with other studies examining systemic treatments in AD [60,61]. While the mean age of patients in the JAK inhibitors group (Group 2) was slightly higher than that of the biologics group (Group 1), the difference was not statistically significant. Interestingly, anthropometric measurements revealed that patients in Group 2 were significantly taller and heavier than those in Group 1. However, these factors were unlikely to play a role in treatment selection, especially when BMI and abdominal perimeter, which are more indicative of overall metabolic health, were comparable between the groups. This is of great importance because, in real-world settings, patients with higher BMI are supposed to be more likely prescribed biologic drugs rather than JAK inhibitors for moderate-to-severe AD, and this could potentially interfere with comparisons between these drugs’ outcomes. Several factors influence this preference, including their differing safety profile. Biologics, such as dupilumab and tralokinumab, are generally considered safer than JAK inhibitors, which have been associated with increased risks of serious adverse events, particularly in populations with higher cardiovascular risk factors [62,63,64].

Residential area type, physical activity, tobacco use, and alcohol consumption did not significantly differ between treatment groups, suggesting that lifestyle factors had minimal influence on the choice of therapy in our area. Most patients resided in urban or semi-rural environments, with only a small proportion living near coastal regions. Notably, while a moderate level of physical activity was observed across the cohort (mean 2.5 exercise sessions per week), no significant association was found between exercise habits and treatment choice. Similarly, the distribution of current, former, and never smokers, as well as alcohol intake, was balanced across the two groups. Next to risk behaviors, a major focus was placed on cardiovascular risk factors. Among them, hypertension proved to be significantly more prevalent in Group 2. This finding disputes the broader understanding of biologic therapies, such as dupilumab, as preferred in patients with comorbid cardiovascular conditions due to their lower immunosuppressive profile and favorable safety record [62,63,64,65]. Conversely, JAK inhibitors, which could be associated with a higher risk of thromboembolic events in some contexts [66], were presumed to be reserved for patients without significant cardiovascular comorbidities. Recently, the authors of an Italian Delphi consensus reviewed the most relevant literature regarding the safety of JAK inhibitors and reached the conclusion that a detailed medical history should be obtained from each patient before starting the medication [67]. These drugs could still be prescribed even to patients over 65 years in the absence of significant risk factors for cardiovascular disease and/or malignancies. Other cardiovascular risk factors in our cohort, including diabetes mellitus, dyslipidemia, and metabolic syndrome, did not differ significantly between groups, suggesting that the overall cardiovascular health status was homogeneously distributed across the sample and thus may not have heavily influenced treatment allocation decisions.

Atopic comorbidities, including asthma, allergic rhinoconjunctivitis, and allergic sensitization, did not differ between groups. Their severity was not assessed, but their high presence is consistent with the shared pathophysiological basis of these conditions and AD, which involves a Th2-skewed immune response [2,68,69,70]. The comparable prevalence of atopic conditions across treatment groups suggests that these comorbidities were not primary determinants in the selection of biologics versus JAK inhibitors for dermatologists in our area. This is striking because biologic therapies that specifically target Th2-skewed cytokines (IL-4, IL-13) are generally thought to have a higher pre-test probability of success in cases of heavier atopic burden [8,62,64,65,71]. Non-atopic comorbidities, such as IMIDs, gastroesophageal reflux, and ophthalmologic conditions, also showed no significant differences between groups. While conditions like psoriasis and alopecia areata share a strong background of immunological activation with AD [12,18,72,73,74,75], their low prevalence in the cohort likely precluded any future impact on treatment outcomes. Additionally, the lack of significant variation in patients with prior VZV infection or reactivation or a history of neoplasms is somewhat unexpected. These conditions are generally considered contraindications or complicating factors for initiating treatment with JAK inhibitors [76]. Reactivations of VZV have been solidly documented in RCTs of JAK inhibitors, underscoring the immunosuppressive effects of these therapies [77,78,79]. Similarly, the broader immunosuppressive profile associated with JAK inhibitors is supposed to limit their use in patients with a recent history of neoplasms due to the potential risk of malignancy recurrence or progression [76]. The lack of differentiation in these two variables, VZV status and oncologic history has different explanations. Regarding the former, it reflects careful patient selection by clinicians and adequate recombinant zoster vaccination protocols. The lack of differences in the latter rather stems from the rarity of recent neoplasms within this cohort.

Baseline disease scores similarity between groups suggests that both cohorts had comparable levels of disease severity and quality-of-life impairment, providing a balanced foundation for comparing treatment outcomes. By week 16, patients in Group 2 seem to have improved more in their quality of life. This is shown by the significant differences between Group 1 and Group 2 scores in severity scales containing subjective reports at week 16. This is consistent with the quicker relief of pruritus that JAK inhibitors achieve compared to biologics. Additionally, another clinically relevant difference observed was the higher number of patients under 18 years of age treated with biologics, reflecting the approved age ranges for these therapies and the longer clinical experience and safety data available for biologic treatments [12]. Despite that, this clinically relevant difference did not reach statistical significance, which might be attained with the constantly larger sample size.

### 4.1. Contextualization with Prior Studies and Clinical Significance

Only scarce evidence is already available regarding real-world uses of biologics versus JAK inhibitors for moderate-to-severe atopic dermatitis. Outcomes comparisons are still in the early phases compared to other IMIDs [80,81]. Some other authors have also paid attention to baseline factors. A study by Schneeweiss MC et al. provides a comparison of baseline characteristics between patients initiating JAK inhibitors (upadacitinib, abrocitinib) and those initiating IL-4/IL-13 inhibitors (dupilumab, tralokinumab). This study found that, after propensity score matching, patient characteristics were well balanced between the groups, indicating that their cohorts were also comparable in terms of demographics and comorbidities [47]. Another population-based cohort study compared patients treated with oral JAK inhibitors (upadacitinib, abrocitinib, and baricitinib) versus dupilumab [80]. Contrary to the previous one, this study found that patients in the JAK inhibitor group were generally younger, more often male, and had a higher prevalence of certain comorbidities such as hypertension and chronic kidney disease compared to those treated with dupilumab. Once again, this opposes our expected pattern of prescription regarding cardiovascular risk factors, considering previously available information. Our findings are different from those described—no notable differences have been observed between treatment groups in our cohort in terms of comorbid profiles—but similarly challenge the established notions and emphasize the variability in real-world treatment patterns. Therefore, we might need to assume the misalignment that exists between the generalized theoretical assumptions—often derived from clinical trials—about distinct disease profiles and how advanced therapies are being utilized in real-world settings. These theoretical assumptions, particularly regarding the presence of atopic comorbidities or cardiovascular risk factors, were derived from structured environments and do not always translate seamlessly into clinical practice. This underscores the need for continued investigation into the practical determinants of treatment decisions beyond theoretical frameworks and controlled trial conditions. It is imperative to continue collecting and analyzing evidence from baseline characteristics in real-world settings. Such data will enable a more nuanced understanding of treatment dynamics to truly evaluate the suitability of each therapy, refining therapeutic strategies according to the needs of diverse patient populations. Joint efforts toward accurately characterizing patients’ profiles across different settings are key to ensuring the extrapolatability of RWD outcomes and comparisons among drugs. The greater homogeneity between groups in our sample is reassuring for subsequent comparisons.

### 4.2. Strengths, Limitations, and Implications for Future Research

Notably, while hypertension was significantly more prevalent in the JAK inhibitor group, the overall cardiovascular risk profiles, based on the variables studied, were comparable between the two groups. The absence of statistically significant differences in most demographics and comorbidities between the two groups is particularly important. This homogeneity in baseline characteristics allows for more meaningful comparisons of treatment outcomes, as potential confounding factors related to group imbalances are minimized. Given the importance of adequately understanding all the characteristics of patients initiating systemic advanced therapies in real-world settings to secure faithful RWD comparisons among drugs, it is impossible to comprehensively address all relevant factors in a single work. This study focused solely on the epidemiological data and comorbidities of patients in our area, leaving other determinants, such as disease-related aspects, unexplored. Baseline disease-related factors (including family and personal history of AD, age of onset, use of emollients, prior treatments received, frequency of emergency room visits, hospitalizations related to AD, and disease clinical pattern…) will be covered in an already envisioned follow-up study to comprehensively evaluate patients’ clinical characteristics at treatment selection. Such research will complement these findings, laying the necessary groundwork for subsequent comparative analyses of treatment outcomes of biologics and JAK inhibitors in our area. This study’s strengths include its real-world design, which captures the diversity and complexity of patients encountered in routine clinical practice. The ambispective nature introduces inherent limitations, such as the potential for missing data or unmeasured confounding variables in retrospective searches. Additionally, while the study cohort provides valuable insights, the sample size reflects that it belongs to a limited geographic area, which may limit the generalizability of findings to other populations or healthcare settings. However, this can also be regarded as a strength for our ongoing research project. The similarity in patient profiles between the two groups, combined with the fact that only a small group of dermatologists were consistently involved in managing these patients, minimizes potential variability in clinical practices that might arise in different regions or healthcare settings, ensuring that no significant differences were overlooked due to geographic, inter-professional or health system disparities. Furthermore, for the goal of comparing advanced therapies in real-life conditions, this focused framework enhances reliability, providing a more solid foundation to build meaningful and accurate comparisons.

## 5. Conclusions

This study provides a detailed characterization of demographics and comorbidities not related to AD in AD patients initiating biologic therapies versus JAK inhibitors in a real-world setting. It represents a step forward in understanding current treatment patterns in our area. While notable differences in hypertension prevalence and anthropometric measures were observed, most baseline characteristics were comparable between the groups. No higher prevalence of cardiovascular risk factors was observed in the biologic group, contrary to what was expected. The only additional clinically relevant difference at baseline between the groups was the higher number of patients under 18 treated with biologics, which aligns with the approved age ranges for each drug and the longer clinical experience with biologic therapies. No differences were found in severity scores at the beginning of the treatment period. This homogeneity in baseline characteristics between groups contributes to enabling future RWD comparisons regarding treatment outcomes in this pool of patients. Other baseline characteristics including further disease-related factors, are first to be addressed if we pursue the generation of a reliable body of evidence that supports holistic and personalized medicine approaches in AD management.

## Figures and Tables

**Table 1:** Main descriptive and inferential statistics from participant demographics and anthropometric measurements.

Variable		Total	Group 1	Group 2	*p*-Value
**Size**	**Sample (absolute number)**	150	102	48	-
**Sex**	**Men (absolute number)**	93	62	31	0.79
	**Women (absolute number)**	57	40	17	0.655
	**Ratio (men:women)**	1.63	1.55	1.82	0.583
**Age**	**Mean ± SD**	35.87 ± 14.37	35.33 ± 15.07	37.00 ± 12.56	0.3627
	**Under 18-years-old**	8	7	1	0.0671 **
**Body**	**Height (mean ± SD [cm])**	170.01 ± 11.15	168.44 ± 11.40	173.71 ± 9.69	0.0066 *
	**Weight (mean ± SD [kg])**	73.06 ± 17.85	71.27 ± 18.81	77.34 ± 14.59	0.0387 *
	**Body Mass Index (mean ± SD [kg/m^2^])**	25.24 ± 4.20	25.08 ± 4.43	25.59 ± 3.66	0.51
	**Abdomen (mean ± SD [cm])**	88.99 ± 13.76	88.58 ± 14.30	89.92 ± 12.55	0.5794

* Statistically significant difference. ** Clinically relevant difference.

**Table 2:** Main *p*-values from contrasts between Group 1 and Group 2 participants’ lifestyle, cardiovascular risk factors, atopic and non-atopic comorbidities.

Variable		Group 1	Group 2	*p*-Value
Physical exercise	No	31	12	0.5261
	Yes	64	31	
Area of residence	Rural	47	20	0.1176
	Urban	43	26	
	Coast	12	2	
Tobacco use	No	63	26	0.4562
	Yes	20	15	
	Former	16	6	
Alcohol intake	No	30	17	0.1761
	Yes	58	28	
	Former	11	2	
Diabetes mellitus	No	100	45	0.4744
	Yes	1	2	
Hypertension	No	99	41	0.0175 *
	Yes	3	7	
Metabolic syndrome	No	99	45	0.604
	Yes	3	3	
Dyslipidemia	No	90	41	0.7723
	Yes	12	7	
Chronic kidney disease	No	102	47	0.6864
	Yes	0	1	
Asthma	No	61	22	0.2144
	Yes	41	26	
Rhinoconjunctivitis	No	34	16	1.0
	Yes	68	32	
Gastroesophageal reflux	No	78	43	0.114
	Yes	23	5	
Pneumoallergens	No	40	19	0.9962
	Yes	62	29	
Trophoallergens	No	78	30	0.1904
	Yes	24	18	
Allergic contact dermatitis	No	70	31	0.6669
	Yes	32	17	
Psoriasis	No	93	41	0.601
	Yes	7	5	
Alopecia areata	No	86	42	0.4881
	Yes	16	6	
Celiac disease	No	101	48	1.0
	Yes	1	0	
Ophthalmologic disease involving red eye	No	91	42	0.9233
	Yes	11	6	
VVZ infection or reactivation	No	16	9	0.7682
	Yes	83	38	
Neoplasm	No	101	48	1.0
	Yes	1	0	

* Statistically significant difference. ** Clinically relevant difference.

**Table 3:** Comparison between Group 1 and Group 2 participants’ severity of disease.

Scale	Group	Week 0 (Mean ± SD)	Week 16 (Mean ± SD)	
EASI	Total	27.55 ± 9.44	7.56 ± 9.20	<0.001 ***
	1	27.15 ± 9.63	6.98 ± 9.53	<0.001 ***
	2	28.43 ± 9.06	8.77 ± 8.37	<0.001 ***
		0.278	0.232	***p*-value**
SCORAD	Total	49.54 ± 14.56	13.42 ± 15.10	<0.001 ***
	1	48.41 ± 14.46	12.61 ± 15.69	<0.001***
	2	51.76 ± 13.85	15.19 ± 14.65	<0.001 ***
		0.9022	0.8211	***p*-value**
DLQI	Total	15 ± 5	8 ± 3	<0.001 ***
	1	16 ± 4	9 ± 2	<0.001 ***
	2	15 ± 6	7 ± 3	<0.001 ***
		0.7214	<0.001 ***	***p*-value**
POEM	Total	22.36 ± 5	7.53 ± 3	**<0.001**
	1	22.15	7.98	**<0.001**
	2	22.65	6.77	**<0.001**
		0.274	0.04 ***	***p*-value**

Abbreviations: EASI: Eczema Area and Severity Index; DLQI: Dermatology Life Quality Index; POEM: Patient-Oriented Eczema Measure; SCORAD: SCORing Atopic Dermatitis. * Statistically significant difference. ** Clinically relevant difference. *** Statistically and clinically relevant difference.

## Data Availability

The data that support the findings are available from the corresponding authors, APC or RRV, upon reasonable request. All the authors had full access to all the data in this manuscript and jointly assume responsibility for its integrity.
